# A critical review of the factors leading to cardiopulmonary resuscitation as the default position of hospitalized patients in the USA regardless of severity of illness

**DOI:** 10.1186/s12245-019-0225-z

**Published:** 2019-03-13

**Authors:** Loukas Georgiou, Anastasios Georgiou

**Affiliations:** 1 Rhodes College, 2000 North Parkway, Box 1641, Memphis, TN 38112 USA; 2 Carl Kirkland Cancer Center, 720 W Forest Avenue, Jackson, TN 38301 USA

**Keywords:** Do-Not-Resuscitate, Cardiopulmonary resuscitation, Presumed consent, Futility, Medicolegal

## Abstract

**Background:**

Physicians are occasionally faced with patients requesting full resuscitation against medical advice. More commonly, neither patients nor their family members make such a request, but physicians simply presume that providing cardiopulmonary resuscitation comports with the patient’s wishes. In the USA, in contrast to other countries, a unilateral Do-Not-Resuscitate order by the physician is either forbidden by State Statute or not enforced by hospital policy. Unless otherwise specified, performing cardiopulmonary resuscitation on all hospitalized patients, regardless of the severity of the underlying illness, is the default position. Unlike other medical interventions, no deference is given to the medical judgment of the physician even when a patient is in the last days of a terminal illness. We examine the factors that have led to cardiopulmonary resuscitation having this unique status.

**Main body:**

A review of the historical factors leading to cardiopulmonary resuscitation as the default position was undertaken. Articles published in the medical literature, lay-press articles, legislative enactments of law, and judicial opinions involving the issue of Do-Not-Resuscitate and cardiopulmonary resuscitation were reviewed regarding their impact on physician and hospital practice in the USA.

**Conclusion:**

A critical review of the historical factors reveals that the rapid dissemination of cardiopulmonary training for the public, inaccuracies in the media regarding successful cardiopulmonary resuscitation, well-meaning legislative efforts with inadvertent consequences, and judicial interpretation outside the generally accepted concept of malpractice law have contributed to the situation faced by today’s physicians and hospitals in the USA.

## Introduction

In comparison with many other countries, physicians in the USA incorporate the patient’s wishes more so in making end of life medical decisions [1–5]. One of the most difficult discussions arises during end-of-life care in which a patient wants cardiopulmonary resuscitation (CPR) against the advice of the physician who feels that such an intervention would be futile—if not detrimental—and argues for a Do-Not-Resuscitate (DNR) status. In the USA, unlike other medical procedures, a hospitalized patient’s consent is needed not to administer CPR. We define a unilateral DNR order as DNR order written by a physician in good conscience but to which the patient has not consented. In the USA, a unilateral DNR order is generally invalid. We have deep misgivings regarding the CPR by default position in these circumstances. Although focusing on the issue in the USA, we feel that the article is still of interest to the international medical community since physicians in other countries face the same issue.

Cardiopulmonary arrest is the end-point of all human life, but not all scenarios are equivalent. Unforeseeable cardiopulmonary arrest due to an allergic reaction is different than the expected death due to terminal cancer. The issue addressed in this review is summarized by the following example. A hospitalized patient is in the last days of life due to progressive metastatic lung cancer. In spite of the patient’s demands, a medical oncologist refuses to administer further chemotherapy. The patient also insists on having CPR in case of a cardiopulmonary arrest. The hospitalist disagrees and bases that decision on the same factors used by the oncologist that this intervention (i.e., administration of CPR) is not appropriate and enters a DNR order. In the majority of hospitals in the USA, the oncologist’s decision would go unquestioned, whereas the hospitalist’s decision would not be supported. Often, there is no discussion regarding CPR status, and by default, the hospitalists will administer CPR on the presumption that the patient would want this intervention fearing the consequences of not administering CPR more than being found wrong and defying the patient’s wishes against CPR. How, then, did CPR achieve this unique status?

There are numerous articles on the evolution and impact of DNR orders [6, 7] and the history of the methods used in CPR [8], with a multitude of books and journals devoted to the bioethics and medico-legal issues of end-of-life care. Admittedly, there are bioethical distinctions between withholding and withdrawing care as medically futile whether on a physiologic or functional bases. However, of all the procedures performed by a physician, few others evoke the degree of emotion associated with CPR. We feel that an examination of how CPR became the default in modern-day medical practice provides a nutshell summary of the larger issues associated with end-of-life care.

A critical review reveals that the need for quick intervention in the out-patient setting coupled with widespread training of the general public in CPR inadvertently led its perception as a panacea. This misperception is reinforced by the media portrayal of CPR. Once this nidus of default CPR had formed, legislative and judicial intervention allowed CPR to become the de facto default position for hospitalized patients even if such intervention is based on broad misconceptions.

## The spread of CPR training

Modern-day CPR has its origins in the operating theater as a surgical intervention. Cardiac compression was initially described in 1628 by Harvey [9], and the concept of internal cardiac massage was first described by Schiff [10] in 1874. The first successful human cardiac resuscitation was performed in 1901 when a patient suffered a cardiac arrest after an abdominal operation at which point the heart was exposed and cardiac compression performed [11]. This technique, however, was limited to highly trained medical professionals and performed most often in the operating room and on patients in good performance status in which the cardiopulmonary arrest was relatively unexpected and a result of an unforeseen complication due to anesthesia. However, in a landmark article published in 1960 in the Journal of the American Medical Association (JAMA), Kouwenhoven et al. reported on a technique using closed chest cardiac massage [12]. By not having to enter the thoracic cavity, this technique could be performed anywhere and taught to a wider group of individuals.

In order to improve on the outcomes, early intervention during cardiopulmonary arrest was advocated. Within a few years, CPR was being implemented by rescue squads in several cities. For example, staff members from John Hopkins Hospital and other Baltimore City hospitals quickly began teaching ambulance and Baltimore City Fire Department rescue personnel closed chest cardiac compression [13]. In another landmark article published in 1967, Irish cardiologist Frank Pantridge et al. reported their model of providing rapid pre-hospital care to patients with an acute myocardial infarction thru a mobile intensive cardiac care unit that was staffed by trained physicians [14]. Thereafter, a similar program in the USA was initiated in the city of New York by St. Vincent’s Hospital. However, physicians were replaced by specially trained personnel referred to as “para-medics” [15]. This model was quickly adopted in several cities including Miami, Columbus, Los Angeles, Portland, and Seattle [16].

In order to minimize the time between cardiopulmonary arrest and performance of CPR, efforts were made to recruit the general public. A program initiated by Dr. Leonard Cobb in Seattle, Washington, was particularly influential. In addition to teaching paramedics pre-hospital cardiac care, the program expanded into being the first in the nation to make citizens part of the emergency system. CPR training was given to citizen volunteers beginning in 1972. Within a few years, it was estimated that 100,000 people had been trained [16].

Numerous victims of cardiac arrest have benefited from citizen training in CPR. The implication is that CPR was to be instituted as rapidly as possible to all individuals. However, in most training seminars, very little time is spent discussing the appropriateness of resuscitation as the emphasis is placed on achieving return of spontaneous circulation (ROSC). A valid argument can be made that the layperson is ill-equipped to determine the appropriateness of initiating CPR. Nonetheless, the impression is left that intervention with CPR is a panacea to be administered to all suffering from cardiopulmonary arrest regardless of the underlying cause of such an arrest.

## The American Heart Association and CPR

Initially, the American Heart Association (AHA) viewed the training of non-medical personnel in CPR with circumspection. In 1962, the AHA stated that CPR was to be applied only by carefully trained physicians [17]. However, in 1965, CPR was classified as an emergency measure to be applied by well-trained physicians, nurses, allied health professionals, and rescue squads [18]. It was felt that the experience with CPR up until that time did not warrant extending training to the general public. In 1973, the AHA finally recommended CPR training for the general public with the announcement being made in the February 1974 issue of JAMA [19].

Initially, the medical literature did address the indications for CPR. The indications were discussed by its modern founders and, initially, straightforwardly in the AHA guidelines. However, this emphasis gradually diminished as the focus centered on teaching the proper resuscitation technique.

It is important to note that Kouwenhoven’s original article primarily involved cardiopulmonary arrest secondary to intra-operative anesthetic complications. Cognizant of this fact, in a 1965 monograph on CPR, one of the original authors of the Kouwenhoven article wrote:

“Resuscitation of the dying patient with irreparable damage to the heart, lungs, brain, or any other vital system of the body has no medical, ethical, or moral justification. The techniques described in this monograph were designed to resuscitate the victim of acute insult, whether it be from drowning, electrical shock, untoward effect of drugs, anesthetic accident, heart block, acute myocardial infarction, or surgery.” [20]

The futility of administering CPR to a dying patient is exemplified in the case of a 68-year-old physician dying of cancer that was published in the British Medical Journal. The 1968 case report clearly recounts how multiple resuscitative attempts by the hospital’s resuscitation team, against the patient’s wishes, resulted in prolonging his agony [21]. Some physicians, however, did question the validity of administering CPR to all individuals regardless of their underlying comorbidities. For example, in a letter to the editor of JAMA entitled “Prevention of Irrational Resuscitation,” a physician took to task what he described as the “new heroism in medicine” which administers CPR to all with cardiopulmonary arrest regardless of their underlying comorbidities [22].

A valid argument can be made that first-responders outside of a hospitalized setting would face tremendous difficulty in determining which individuals would not benefit from CPR. In addition, the medical literature clearly supports that early intervention for certain cardiac arrhythmias improves the chance of successful resuscitation. Therefore, it is entirely understandable to give individuals suffering out-of-hospital cardiopulmonary arrest (OHCA) the benefit of the doubt and make intervention with CPR the default position. However, these same arguments are meritless when individuals have arrived in an emergency department and have been fully evaluated. This is especially true in the non-emergency setting in which a terminally ill inpatient demands CPR despite medical evidence showing no benefit.

Of interest is the evolution with time of the view espoused by the AHA regarding the administration of CPR. It is noted that the AHA is the largest organization responsible for CPR training and certifying health professionals in the USA. With time, the organization’s recommendations progressed from a more restrictive to a more troubling less restrictive view in determining who would benefit from CPR. For example, in 1997, in the chapter entitled “Ethics of CPR and Emergency Cardiovascular Care (ECC)”, unilaterally withholding or terminating resuscitation was justified under the principle of medical futility when ongoing resuscitative measures were attempted with no ROSC, a patient’s vital functions continued to deteriorate despite maximal therapy, or when no survivors have been reported to survive to discharge in well-controlled trials [23]. The guideline recommended that the patient or the patient’s surrogates should be informed of the no-CPR order but not offered the choice of CPR [23].

However, in the 2010 guidelines, a distinction was made between OHCA and the in-hospital cardiac arrest (IHCA). The guidelines for IHCA were as follows:

Few criteria can accurately predict the futility of continued resuscitation. In light of this uncertainty, all pediatric and adult patients who suffer cardiac arrest in the hospital setting should have resuscitative attempts initiated unless the patient has a valid DNAR [Do Not Attempt Resuscitation] or has objective signs of irreversible death (e.g. dependent lividity). [24]

Unfortunately, this guideline is not helpful to physicians who must weigh the benefits versus the risks for any procedure knowing that the outcome of many other interventions can be predicted to degree equivalent to the outcomes of CPR. Studies consistently show that the probability of successful resuscitation is heavily dependent on the patient’s overall medical condition. Underlying malignancy [25–27], coexisting chronic health conditions [25, 28, 29], coexisting organ failure [29, 30], need for vasopressor support [31, 32], prior need for CPR [33, 34], and advanced age [25, 35–37] all bode for a very poor outcome with successful CPR almost universally measured in the single digits.

The AHA 2015 Guidelines regarding not starting CPR in IHCA are much harder to interpret. Under a heading entitled “Withholding and Withdrawing CPR (Termination of Resuscitative Efforts) Related to In-Hospital Cardiac Arrest,” the following wording is used:

In the 2010 Guidelines, it was noted that not initiating resuscitation and discontinuing life-sustaining treatment of in-hospital cardiac arrest (IHCA) during or after resuscitation are ethically equivalent, and clinicians should not hesitate to withdraw support on ethical grounds when functional survival is highly unlikely. [38]

It is difficult to ascertain whether it is withholding of CPR or withdrawal of CPR after it is instituted that is ethical. The reader is left with the impression that withholding of CPR may be entirely appropriate but the AHA is hesitant to explicitly to state so in the guidelines. In contrast, the European recommendations have consistently addressed the ethics of withholding intervention due to medical futility [39, 40]. Guidelines in Australia [41] and New Zealand [42, 43] have similar considerations. Table 1 summarizes the position taken by the AHA throughout the last two decades.

**Table 1:** American Heart Association guidelines on not administering CPR

Year	Guideline
1997	Medical futility justifies unilateral decisions by physicians to withhold or terminate resuscitation under the principle of medical futility. [23]
2010	“All pediatric and adult patients who suffer cardiac arrest in the hospital setting should have resuscitative attempts initiated unless the patient has a valid DNAR [Do Not Attempt Resuscitation] or has objective signs of irreversible death (e.g. dependent lividity).” [24]
2015	“In the 2010 Guidelines, it was noted that not initiating resuscitation and discontinuing life-sustaining treatment of in-hospital cardiac arrest (IHCA) during or after resuscitation are ethically equivalent, and clinicians should not hesitate to withdraw support on ethical grounds when functional survival is highly unlikely.” [38]

## The media and public misperception of the benefits of CPR

Studies show that most laypeople significantly over-estimate the chance of survival for individuals that undergo resuscitation after a cardiac arrest [44]. Television and public programs as a source of information regarding CPR are factors identified as leading to unrealistic expectation of survival [45].

The outcomes of CPR conveyed by most television medical dramas in the USA and in Europe are much higher than what is reported in the medical literature [46–48]. Even if the initial outcomes of CPR are accurately portrayed, the long-term outcomes of resuscitation are not portrayed and therefore give a false expectation [49]. For example, a review of the television shows Chicago Hope, ER, and Rescue 911 noted a 65% overall survival rate for CPR with a 67% survival to discharge rate [50]. On the contrary, the medical literature reports rates of hospital discharge as consistently less than 20% [51]. Given this significant misunderstanding regarding the benefits of CPR, it is not surprising that studies show that discussions between physicians and patients only result in a small benefit in altering a patient’s choice regarding the desire for CPR [52–55].

## The Legal Quagmire regarding CPR and DNR orders

As a consequence of the factors noted above, it was inevitable that legislators and the judiciary in the USA would intervene in the subject resulting in laws that directly or indirectly affect the issue of CPR. Therefore, the effect of a limited set of landmark laws and judicial decisions is warranted. With the exception of the New York Statute, the rulings and laws to be discussed do not explicitly address the issue of CPR. Also, it is noted that no law or ruling in the USA demands that physicians administer futile care—although an argument to this effect can be made regarding the Emergency Medical Treatment and Active Labor Act (EMTLA).

Nonetheless, the authors note that almost every major hospital has an ethics committee whose purpose is to resolve patient-physician conflicts that center around end-of-life care of which CPR status is one component. If the placement of a unilateral DNR in good conscience was without repercussion, then at least that portion of the deliberation conducted on the ethics committee would become irrelevant. From personal experience as a practicing oncologist for 25 years, one of the authors has been to numerous deliberations in which the need for an ethics consult arose as a result of the consequence of a physician not placing a unilateral DNR (i.e., a severely ill patient with a terminal disease was resuscitated but now the physician and hospital find themselves administering futile care to a patient who is comatose, intubated, and with multi-system organ failure in which—once again—the physicians are not willing to enter a unilateral DNR order and the hospital is not willing to support such an order) but has never seen an ethics consult entered due the refusal of a physician to administer further chemotherapy to such a patient.

Therefore, even if it is a misperception of the law, a physician not acquiescing to a patient’s demand for CPR will face professional difficulties. In the USA, placing a unilateral DNR would most likely result in a review by the hospital administration and, at a minimum, threats of legal challenges. Even if the physician was not held legally liable, the frustration of dealing with an investigative body, the protracted legal process, and the bad publicity would make it a Pyrrhic victory.

Initially, the legal issues centered on the timing of death. Historically, cessation of spontaneous cardiopulmonary function was the hallmark of death. In the actively dying patient, CPR usually delayed but did not halt the dying process. In their 1965 monograph, in the first chapter entitled “Essentials,” two of the cofounders of modern-day CPR clearly stated that the first principle to be observed is that:

The patient must be salvable. Cardiopulmonary resuscitation is indicated for the patient who, at the time of cardiopulmonary arrest, is not in the terminal stage of an incurable disease. Resuscitative measures on terminal patients will, at best, return them to the dying state. [56]

Unfortunately, such advisories carried no legal weight and were of no defense when prosecutors began to view withholding or withdrawing life support as hastening the time of death. Following the letter rather than the spirit of the law, charges of homicide were threatened or even brought against physicians. One of the earliest cases involved two physicians in California who were prosecuted for murder by the District Attorney in the City of Los Angeles after removing, with the family’s consent, a comatose patient from ventilator support and subsequently removing the nasogastric tube used for artificial feeding [57]. Fortunately, the Superior Court for the State of California dismissed the case and ruled in favor of the physicians with the following statement:

A physician has no duty to continue treatment, once that has proved to be ineffective. Although there may be a duty to provide life-sustaining machinery in the immediate aftermath of a cardio-respiratory arrest, there is no duty to continue it use once it has become futile in the opinion of qualified medical personnel. [57]

Although the case was resolved in the physician’s favor, the chilling effect had repercussions. With the exception of the United States Supreme Court, there is no unifying judicial authority in the USA. Therefore, there was no guarantee that other jurisdictions would not follow this precedent of filing charge of homicide. As will be discussed later, physicians in at least one other State (i.e., the State of New York) took note of this possibility.

As a general rule, current case law makes it improbable that a physician will be held liable for homicide for withholding futile care. However, it is stressed that there is no universally established case law directly addressing the issue of withholding CPR on the basis of futile care. On the contrary, it seems that the issue of CPR has achieved a special status to which arguments of futile care do not apply. As discussed below, a physician entering a unilateral DNR on grounds of futility is steering a course for very murky waters. In the State of New York, these waters are forbidden.

## The Patient Self-Determination Act

Similar to physician misperception, legal intervention led to misconceptions on the patient’s behalf regarding the ability to demand treatment. Ironically, this arose as a result of physicians’ intervening against a patient’s desire for intervention. It should be noted that to some degree, this behavior on the physician’s part was fueled by not wishing to run afoul of unsettled law at that time with prosecutors becoming involved in some of the more famous right-to-die cases. The ability of physicians to keep patients alive even with severe functional deficits eventually led to a constitutional struggle between the state’s right to protect life and the patient’s right to refuse treatment. Ultimately, the courts accepted that a patient had a right to refuse medical life-sustaining treatment even if that refusal led to death [58–60].

In 1990, this prompted passage of the Patient Self Determination Act (PDSA) that requires hospitals to inform patients of their right to refuse CPR and other measures [61]. However, a patient’s right to refuse CPR is not tantamount to a patient having a right to demand CPR. By analogy, a patient entering the hospital for a bowel obstruction may refuse surgical intervention, but it is not assumed that he has the right to demand such intervention if it is not appropriate. Unfortunately, the issue of CPR began to be addressed as a simple yes or no choice without the caveat that, as with every other medical procedure, it would be offered only if deemed medically appropriate.

A bioethicist nicely summarizes the situation thusly:

The present emphasis on autonomy and patient self-determination has led to the belief that patients or proxies have the right to whatever life-sustaining intervention they desire. [62]

## The Emergency Medical Treatment and Active Labor Act

Although not addressing the issue directly, EMTALA and its subsequent interpretation by the courts had a profound impact on the end-of-life care of which CPR is an integral part. EMTALA requires hospitals to give emergency aid to patients who suffer from an “emergency medical condition.” Congress enacted EMTALA in order to prevent hospitals from dumping patients, who lack insurance, by either refusing treatment or transferring them to other hospitals [63].

In the now infamous *Baby K* case, the United States District Court for Eastern Virginia interpreted EMTALA as requiring stabilization, which may include intubation and full cardiopulmonary resuscitation, even if this is outside the medical standard of care. The Court interpreted the “plain language” of EMTALA as requiring stabilizing treatment for any individual who comes to a participating hospital and is diagnosed as having an emergency medical condition [63]. The Court specifically swept aside any arguments regarding the standard of care by stating that “EMTALA *does not* [emphasis added] provide an exception for stabilizing treatment physicians may deem medically or ethically inappropriate.” [64] However, any semblance of the “plain reading” doctrine of EMTALA was disregarded by the 6th Circuit Court of Appeals in deciding *Thornton v. Southwest Detroit Hospital* [65]. In its ruling, the Court held that EMTALA applies to inpatients as it interpreted “emergency room care” to mean any “emergency care.” [66] Taken at face value, this would mean that any IHCA required resuscitative efforts.

Paradoxically, the “plain reading” of EMTALA was not so plain after all as the 4th Circuit Court of Appeals [67], 9th Circuit Court of Appeals [68], and the 11th Circuit Court of Appeals [69] had ruled that EMTALA did not apply to inpatients. In 2003, the Centers for Medicare and Medicaid Services (CMS) issued regulations expressly clarifying that EMTALA ceases to apply when an individual is admitted to the hospital [70]. Therefore, the older rulings applying EMTALA to inpatients are not currently valid.

However, the chilling effect of the rulings clearly came across to hospitals and physicians as legislated standards of care. This is best summed up by the following from the American Academy of Emergency Medicine:

Initially, the law was enacted to stop patient “dumping.” However, over time, EMTALA has become what it is today—a federally mandated standard of practice for “participating” hospitals (those that have a Medicare provider agreement) and “any physician who is responsible for the examination, treatment, or transfer of an individual in a participating hospital including a physician on-call for such an individual.” [71]

## New York State Legislation

New York was the first state to pass legislation addressing the issue of CPR and DNR. The task force behind the initial statute explicitly recognized physician judgment in entering a unilateral DNR order. The statute itself remained silent on the issue. However, prosecutorial zeal transformed its interpretation into mandating that CPR be the default position.

As discussed above, prior to passage of the legislation, the fear of prosecution for withholding CPR had an impact on physicians which unfortunately led to DNR orders becoming covert in their nature. As an example, in order to avoid a permanent record of the decision, physicians placed removable purple decals on the nursing records of critically ill patients at LaGuardia Hospital in Forest Hills, NY, signifying that attempts at resuscitation were not to be performed [72]. The fact that physicians took these measures, when in good faith it was felt that withholding efforts at CPR was medically appropriate, points to the fact that CPR as a default position had already become established to some degree in the public’s mind.

In light of such behavior, the State of New York convened the New York State Task Force on Life and the Law that proposed legislation that affirmed the presumption that all patients consent to CPR in case of a cardiopulmonary arrest. Despite this presumption for CPR, the Task Force’s recommendation did explicitly state that a DNR order could be entered by a physician if the patient was terminally ill or resuscitation would only serve to prolong the dying process [73]. Consequently, in July 1987, the New York State Legislature amended the State’s public health care law regulating DNR orders. However, the Statute did not address the issue of physicians entering a unilateral DNR on the grounds of medical futility [74].

In 1992, in order to help physicians interpret the new law, a pamphlet was issued by the New York State Department of Health, the New York State Task Force on Life and the Law, the Medical Society of the State of New York, and the Hospital Association of New York State which clearly supported the placing a unilateral DNR in cases of medical futility as judged by two independent physicians. In a pamphlet titled “Do-Not-Resuscitate Orders: Questions and Answers for the Health Care Professionals (2d ed. 1992)” [74], the following question was addressed:

Q: What if the health care agent or surrogate refuses to consent to a DNR order and the physician believes that CPR would be futile for the patient?

The attending physician must seek a second opinion. If the second physician concurs that CPR will be futile, as futility is defined by the law, and the concurrence is written in the chart, the attending physician may enter the order on grounds of futility, but must inform the agent or surrogate.

However, in 2003, in a formal opinion rendered by then New York State Attorney General Eliot Spitzer, New York State Public Health law §2965 was interpreted as prohibiting the placement of a unilateral DNR without the physician obtaining a court order (https://ag.ny.gov/sites/default/files/opinion/2003-F1%20pw.pdf). The New York Attorney General attempted to ameliorate the impact of such an interpretation by noting that after an arrest occurs and resuscitation is underway, the New York Statute does not apply and that a finding of “futility will justify a decision to forego resuscitation” [75].

Attorney General Eliot Spitzer’s interpretation results in an interesting scenario. The decision to terminate resuscitative efforts is left to the learned discretion of the physicians and any disagreement in the medical management during resuscitation would be, presumably, argued under the medical malpractice rubric. On the other hand, the very same physician’s judgment on whether to initiate resuscitation is overruled by the State’s legislature. This quixotic scenario is not unique to American jurisprudence. Along similar lines, the Supreme Court of Canada recently wrestled with a similar decision resorting to a dubious distinction between withdrawing and withholding care [76]. This logical impasse would have been avoided if the legislature and the courts viewed CPR as a medical procedure and analyzed its application under established medical malpractice law.

## Conclusion

In summary, as the application of CPR diffused outside the hospital setting, the endpoint was to emphasize proper technique. Placing the outcome in broader context of choosing the appropriate candidates that would benefit from CPR did not receive similar emphasis. In retrospect, this may have been inevitable as widespread use of CPR for out-of-hospital cardiac arrests is a worthwhile goal. However, the unwanted by-product was that CPR became to be seen as a panacea. In addition, unlike every other medical intervention which is judged on the merits of traditional malpractice law with medical professionals delineating the standard of care, legislatures and courts have imposed their own standards.

## Abbreviations

ACLS: Advanced cardiac life support
AHA: American Heart Association
BLS: Basic life support
CPR: Cardiopulmonary resuscitation
DNR: Do-Not-Resuscitate
ECC: Emergency cardiovascular care
EMTALA: Emergency Medical Treatment and Active Labor Act
IHCA: In-hospital cardiac arrest
JAMA: Journal of the American Medical Association
OHCA: Out-of-hospital cardiac arrest
